# Human basonuclin 2 up-regulates a cascade set of interferon-stimulated genes with anti-cancerous properties in a lung cancer model

**DOI:** 10.1186/s12935-017-0394-x

**Published:** 2017-02-06

**Authors:** Egon Urgard, Anu Reigo, Eva Reinmaa, Ana Rebane, Andres Metspalu

**Affiliations:** 1 Department of Biotechnology, Institute of Molecular and Cell Biology, University of Tartu, Tartu, Estonia; 2 Estonian Genome Center, University of Tartu, Tartu, Estonia; 3 Institute of Biomedicine and Translational Medicine, University of Tartu, Tartu, Estonia; 4 Department of Immunoanalysis, United Laboratories, Tartu University Hospital, Tartu, Estonia

**Keywords:** BNC2, Type I IFN, XAF1, OAS family, Lung cancer

## Abstract

**Background:**

Human basonuclin 2 (BNC2) acts as a tumor suppressor in multiple cancers in an as yet unidentified manner. The role and expression of the *BNC2* gene in lung cancer has not yet been investigated.

**Methods:**

BNC2 expression was studied in the A549 and BEAS-2B cell lines, as well as in lung cancer tissue. Illumina array analysis and a viability assay were used to study the effects of transient transfection of BNC2 in A549 cells. Ingenuity pathway analysis and g:Profiler were applied to identify affected pathways and networks. RT-qPCR was used to validate the array results.

**Results:**

We showed the reduced mRNA expression of BNC2 in non-small cell lung cancer tissue and lung cancer cell line A549 compared to non-cancerous lung tissue and BEAS-2B cells, respectively. Further array analysis demonstrated that the transfection of BNC2 into A549 cells resulted in the increased expression of 139 genes and the down-regulation of 13 genes. Pathway analysis revealed that half of the up-regulated genes were from the interferon/signal transducer and activator of transcription signaling pathways. The differential expression of selected sets of genes, including interferon-stimulated and tumor suppressor genes of the *XAF1* and *OAS* families, was confirmed by RT-qPCR. In addition, we showed that the over-expression of BNC2 inhibited the proliferation of A549 cells.

**Conclusion:**

Our data suggest that human BNC2 is an activator of a subset of IFN-regulated genes and might thereby act as a tumor suppressor.

**Electronic supplementary material:**

The online version of this article (doi:10.1186/s12935-017-0394-x) contains supplementary material, which is available to authorized users.

## Background

Lung cancer is the most malignant tumor and the leading cause of cancer deaths worldwide, with 1.8 million new cases in 2012 [1]. In Estonia, the incidence rate for lung cancer per 100,000 was 71 for men and 14 for women in 2012 [2]. Non-small cell lung cancer (NSCLC) accounts for 80–85% of all lung malignancies. In contrast to the steady increase in survival for most cancers, advances have been slow for lung cancer, with a corresponding 5-year relative survival of 18% [3]. Depending on the stage of cancer, treatment options for people with NSCLC include surgical resection, chemotherapy, radiation therapy, targeted therapy and immunotherapy [4, 5]. Increasing focus has been placed on the development of immunotherapies, including the directed targeting of specific immune suppressors, such as cytotoxic T-lymphocyte antigen-4 protein (CTLA-4) and programmed cell death-1 protein receptor (PD-1) [5–7]. Another important group of immunotherapeutics have been developed based on interferons (IFNs). IFNs are naturally occurring cellular cytokines that activate immune responses and have been shown to have anti-proliferative, anti-angiogenic and pro-apoptotic effects [8, 9].

IFN receptor signaling induces the up-regulation of many ISG-s (interferon stimulated genes), including genes with antiviral properties, such as protein kinase R (*PKR*), 2,5-oligoadenylate synthetase (*OAS*) and myxovirus resistance protein (*MX*) family genes [10–14]. In addition to the ISG-s implicated in anti-viral, anti-angiogenic, immunomodulatory and cell cycle inhibitory effects, oligonucleotide microarray studies have identified ISG-s with apoptotic functions, such as XIAP associated factor-1 (*XAF1*), caspase-4, caspase-8, death activating protein kinases (*DAPKs*) and *IRF*s [15–18].

Human BNC2 is an evolutionarily conserved C2H2 zinc finger protein, which has been suggested to be involved in the regulation of mRNA splicing, processing [19, 20] or transcription [19–21]. BNC2 has been detected in a wide range of tissues: it is abundantly expressed in the ovary, skin, uterus, and kidneys, and its expression has also been detected in the testis, prostate, and lung [19, 20, 22]. BNC2 expression has been detected in cell lines, including primary human keratinocytes, the keratinocyte cell line HaCaT, and HeLa and HEK293 cells [19].

Little is known about the expression and function of BNC2 in tumor progression. Genetic variations in the *BNC2* gene have been associated with skin cancer risk [23–25], susceptibility to ovarian cancer [26–28] and prostate cancer development [29, 30]. The deletion of the *BNC2* gene and the corresponding decreased expression of BNC2 mRNA have been detected in Barrett’s esophagus [31], hepatocellular carcinoma [32] and high-grade serous ovarian carcinoma [33]. In esophageal adenocarcinoma cells, the stable expression of BNC2 caused the growth arrest of tumor cells [31], suggesting that *BNC2* might also be a tumor suppressor gene. Thus far, there is no evidence of the role of BNC2 in lung cancer.

In this study, the mRNA expression of BNC2 was analyzed in lung squamous cell carcinoma tissue samples and a lung cancer cell line. In addition, the effect of transfected BNC2 on gene expression and cell viability was investigated in the human lung carcinoma cell line A549.

## Methods

### Tumor samples

Lung squamous cell carcinoma (SCC) and corresponding adjacent non-tumor tissue samples were collected from 8 patients who had undergone curative resection and been histologically characterized by a clinical pathologist in Tartu University Lung Hospital (Tartu, Estonia). The study was approved by the Research Ethics Committee of the University of Tartu, and written informed consent was obtained from all patients. Tissue specimens of appropriate sizes (1–2 cm^3^) were cut from tumorous and morphologically tumor-free lung tissue. One half of each sample was fixed in formalin and used for pathological examination. The other half of each specimen was snap frozen and stored at −80 °C until use.

### Cell culture

The adenocarcinomic human alveolar basal epithelial cell line A549 and human normal lung epithelial cell line BEAS-2B were purchased from the American Type Culture Collection (Manassas, VA, USA). A549 cells were grown in RPMI-1640 medium (PAA Laboratories, Linz, Austria) supplemented with 10% fetal bovine serum (FBS) (Biochrom AG, Berlin, Germany) and penicillin/streptomycin (PAA Laboratories, Linz, Austria). BEAS-2B cells were grown in DMEM (Lonza, Cologne, Germany) medium supplemented with 3% FBS (Biochrom AG, Berlin, Germany) and penicillin/streptomycin (PAA Laboratories, Linz, Austria). Both cell lines were cultured in a humidified tissue culture incubator with 5% CO_2_ at 37 °C.

### Plasmids and transfections

The expression plasmid containing full-length human BNC2 coding sequence and corresponding empty plasmid pCMV-HA (https://www.addgene.org/32530/) were kindly provided by Dr. Satrajit Sinha (State University of New York, NY, USA). For transient transfection, 10^6^ A549 cells were electroporated with 5 µg plasmid DNA in 250 µl Ingenio electroporation solution (Mirus Bio LLC, Madison, WI, USA) using the Gene Pulser Xcell Electroporation System (Bio-Rad, Stockholm, Sweden) under the following conditions: 280 V, 950 µF and ∞ Ω. After electroporation, cells were plated and harvested every 24 h for 3 days.

### Cell viability assay

For the viability assay, 2 × 10^4^ A549 cells per well were seeded in a 24-well plate. The next day, cells were transfected with expression plasmid containing a full-length human BNC2 coding sequence and corresponding empty plasmid pCMV-HA using Lipofectamine 2000 (Invitrogen, Carlsbad, CA, USA) according to manufacturer’s instructions. Cell proliferation was measured 48 h after transfection using CellTiter-Glo^®^ Luminescent Cell Viability Assay (Promega, Madison, WI, USA), where the Luciferase activity was proportional with the quantity of cellular adenosine triphosphate (ATP).

### RNA extraction and RT-qPCR

Total RNA was isolated using the Ambion RNA extraction kit (Ambion Inc., Austin, TX, USA) according to the manufacturer’s instructions. One microgram of total RNA was converted to cDNA using the First Strand cDNA Synthesis kit (Fermentas, Vilnius, Lithuania). Real-time PCR was performed using SYBR Green ROX mix (Fermentas, Vilnius, Lithuania) and ABI 7900HT Sequence Detection System (Applied Biosystems, Foster City, CA, USA). Data were analyzed using SDS 2.2.2 software (Applied Biosystems, Foster City, CA, USA). The primer sequences for RT-qPCR amplifications are shown in Table 1. Gene expression levels were determined by the 2^−ΔΔCT^ method [34] after normalization to ESD (Esterase D) [35]. Relative gene expression was calculated as a fold change compared to the control transfection.

**Table 1:** List of oligonucleotide primers

Gene	Forward primer	Reverse primer
Esterase D (ESD)	ATTTGCTCCAATTTGCAACC	TCACAAGGTGGGTAGCATCA
Basonuclin 2 (BNC2)	TGTGAAACTTCACTACAGGAACG	GAGGCGTCTTCCCTGACATC
Guanylate binding protein 1, interferon-inducible (GBP1)	CCAGATGACCAGCAGTAGAC	AAGCTAGGGTGGTTGTCCTT
Myxovirus (influenza virus) resistance 2 (mouse) (MX2)	TGAGTGCTGTGTAAGTGATGG	GGACCGGCTAACAGTCACTA
2′-5′-oligoadenylate synthetase 2, 69/71 kDa (OAS2)	GGTAGCGCATCTTGATTCCA	GAGTATGTAGGGTGGCAAGC
Interferon regulatory factor 7 (IRF7)	ATCTTCAAGGCCTGGGCTG	CAGCGGAAGTTGGTTTTCCA
Interferon induced transmembrane protein 1 (IFITM1)	CTGCAACCTTTGCACTCCA	TGTAGACAGGTGTGTGGGTA
Aconitase 1, soluble (ACO1)	GCTCACAGGGCAAGAACGAT	TCATGACAGCCTGGAAGGTC
Differentiation antagonizing non-protein coding RNA (DANCR)	ACTATGTAGCGGGTTTCGGG	TTCCGCAGACGTAAGAGACG
Leucine rich repeat containing 20 (LRRC20)	CTGCTTGGAGAGTTTGCCCT	GCTTAGGGGCTCACTCACTG
5′-nucleotidase domain containing 2 (NT5DC2)	CATCTTCCGCACCTTCCACA	TGAAGTCCACGCGGTAGTTG
Thioredoxin domain containing 12 (endoplasmic reticulum) (TXNDC12)	GCTTGAGCTTCCCTGTTTGC	TGGCTACACCTAGGGCTTGA
2′-5′-oligoadenylate synthetase 1, 40/46 kDa (OAS1)	CGGACCCTACAGGAAACTTG	GAGGTCTCACCAGCAGAATC
2′-5′-oligoadenylate synthetase 3, 100 kDa (OAS3)	AGAGTTCTGAGCAGGGCCTA	TGGAAAGAGCCACCTAACTGC
2′-5′-oligoadenylate synthetase-like (OASL)	ATTCCAAGGCCAAGTCCTG	TCTTCGAGAGGATGAGAGTGT
XIAP associated factor 1 (XAF1)	GGTTTGCCCAAGGACTACAA	GGGTGTAGGATTCTCCAGGT

### Array analysis

The Illumina^®^ TotalPrep™ RNA Amplification Kit (Ambion Inc., Austin, TX, USA) was used to generate biotinylated amplified RNA for hybridization with Illumina HumanHT-12 v4 Expression BeadChip (Illumina Inc., San Diego, CA, USA) and the Illumina BeadChip platform (Illumina Inc., San Diego, CA, USA). Experiments were performed according to the manufacturer’s instructions. Raw expression data were collected and background subtracted by Illumina GenomeStudio Gene Expression Module v1.8.0 (Illumina, Inc., San Diego, CA, USA). Data were transformed by variance-stabilizing transformation and quantile normalization using the Lumi package (v 2.14.0) [36] from Bioconductor (https://www.bioconductor.org/). Differentially expressed genes were identified using the Limma package (v 3.18.1) [37]. A *p* value of 0.05 was used as threshold for differential expression after multiple testing correction by the Benjamini-Hochberg method [38].

### Gene enrichment analysis

Pathway and gene ontology (GO) enrichment analyses were performed with ingenuity pathway analysis (IPA) Ingenuity Systems (http://www.ingenuity.com) (Qiagen, Redwood City, CA, USA) and g:Profiler (http://biit.cs.ut.ee/gprofiler/) [39] using the default settings and the g:SCS method for statistical analysis. The g:SCS method computes multiple testing corrections for *p* values from GO and pathway enrichment analysis using a threshold of 0.05. All reported pathways and biological processes are listed according to their GO enrichment score provided by the two software packages as −log (*p* values) and with a false discovery rate (FDR) <0.05%.

### Statistical analysis

Statistical significance between the different groups and conditions was assessed with Student’s *t*-test, the Wilcoxon matched pair test was used to analyze the relative mRNA expression in tumor and matched adjacent non-tumor tissues. Results were considered significant at *p* < 0.05 (*) and highly significant at *p* < 0.01 (**). Statistical analysis was performed using GraphPad Prism5 (GraphPad Software, San Diego, CA, USA).

## Results

### Decreased expression of BNC2 in the lung carcinoma cell line and in lung cancer tissue

**Fig. 1:**
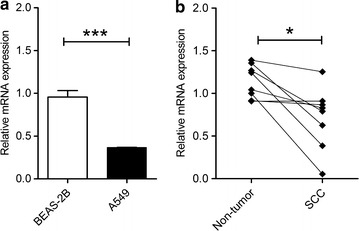
The expression of BNC2 is reduced in the lung carcinoma cell line A549 and lung cancer tissue. **a** Relative BNC2 mRNA expression in A549 and BEAS-2B cell lines. The mean expression of BNC2 in BEAS-2B cells was normalized to 1. Data represent the mean ± SEM from four transfections. Student’s *t*-test, ***p value < 0.001. **b** Expression of BNC2 in 8 pairs of squamous cell carcinoma (SCC) and adjacent non tumor tissues (NL). Wilcoxon matched pair test, *p value <0.05

To study whether the expression of BNC2 is altered in lung cancer, similarly to other tumors [26, 31–33], first, RT-qPCR was used to analyze the mRNA expression level of BNC2 in the human adenocarcinomic alveolar epithelial cell line A549 and the normal lung epithelial cell line BEAS-2B. As shown in Fig. 1a, significantly lower expression levels of BNC2 were detected in the A549 cell line compared to BEAS-2B cells. Next, we tested the expression of BNC2 in 8 pairs of matched SCC and adjacent non-tumor tissues. Consistent with the results in the A549 cell line, the BNC2 expression level in cancerous SCC tissues were lower than in corresponding non-tumor tissues (Fig. 1b).

### Transient transfection of BNC2 affects cancer cell proliferation and global gene expression patterns

**Fig. 2:**
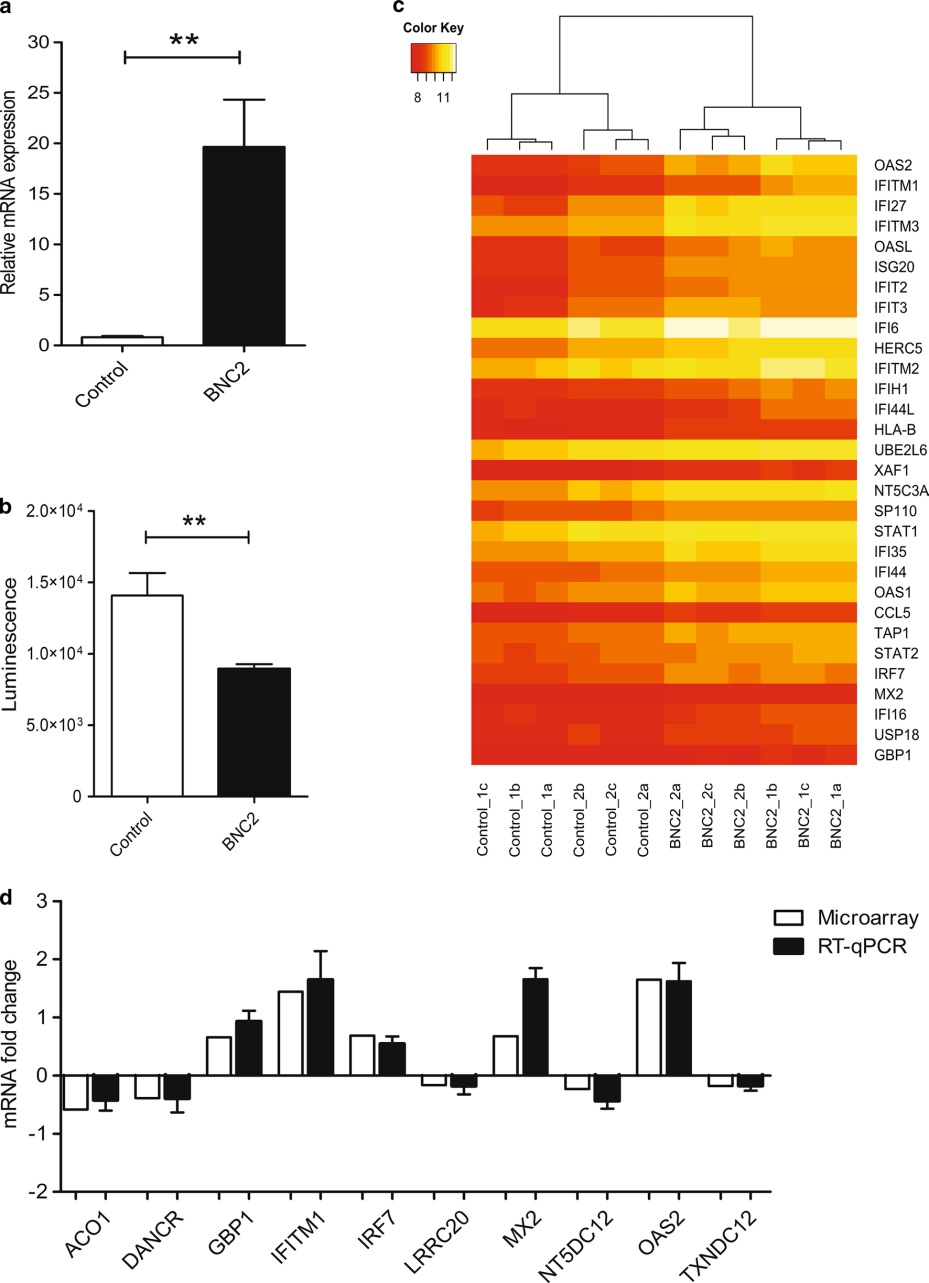
The effect of BNC2 transfection in A549 cells. **a** Relative BNC2 mRNA expression was measured 48 h after the transfection. Human A549 cells were transfected with either BNC2 expression vector or the control (empty vector). Data represent the mean ± SEM of four transfections. Student’s *t*-test, **p value <0.01. **b** The proliferation rate of A549 cells was measured 48 h after transfection. A549 cells were transfected with either BNC2 expression vector or the control (empty vector) followed by Luminescent Cell Viability Assay. Student’s *t*-test, **p value <0.01. **c** Heatmap of the top 30 of the most differentially expressed genes from array data chosen based on fold changes. *Red* represents the lower and *yellow* the higher expression of each gene in all six samples. Data represents the quantile normalized expression values across all of the samples in heatmap. The column-side dendrogram represents the hierarchical clustering of control and BNC2-transfected samples using the complete linkage method with Euclidean distance measures. The samples were collected from two sets of transfections performed in triplicate both times. **d** Comparison of microarray and RT-qPCR results. Data are normalized to the control-transfected cells and are shown as a log2-transformed mRNA fold change. The RT-qPCR results represent four independent transfections with the error bar indicating SEM

Although the reduced expression of BNC2 has been detected in several tumors [26, 31, 32], the role of BNC2 in the suppression of the cancerous processes in the lung has not been studied before. Thus, we analyzed whether BNC2 over-expression has an effect on the cell proliferation rate and global gene expression pattern in the A549 cell line. A greater than 20-fold increase in BNC2 mRNA expression was detected in A549 cells transfected with BNC2-coding plasmid (Fig. 2a), which led to the reduced proliferation rate of A549 cells compared to the control after 48 h of transfection (Fig. 2b).

Next, total RNA samples from both conditions were harvested and subjected to Illumina Expression BeadChip containing 47,000 probes for more than 31,000 annotated genes. Out of the more than 24 000 expressed genes (detection *p* < 0.05), 152 genes (195 transcripts) were altered in response to BNC2 over-expression (139 genes (181 transcripts) up-regulated, 13 genes (14 transcripts) down-regulated). The heatmap in Fig. 2c contains the top 30 genes with the largest fold change. The full list of all significantly changed genes (*p* < 0.05) is provided in Additional file 1. A set of the differentially expressed genes was then validated by means of RT-qPCR. For the validation, we chose two strongly up-regulated genes, *OAS2* and *IFITM1*, and randomly selected three other up-regulated and five down-regulated genes. Although some variation in the extent of fold changes was observed, the RT-qPCR analysis substantially confirmed the results of microarray analysis (Fig. 2d).

### Pathway and network analysis of BNC2-influenced genes

**Fig. 3:**
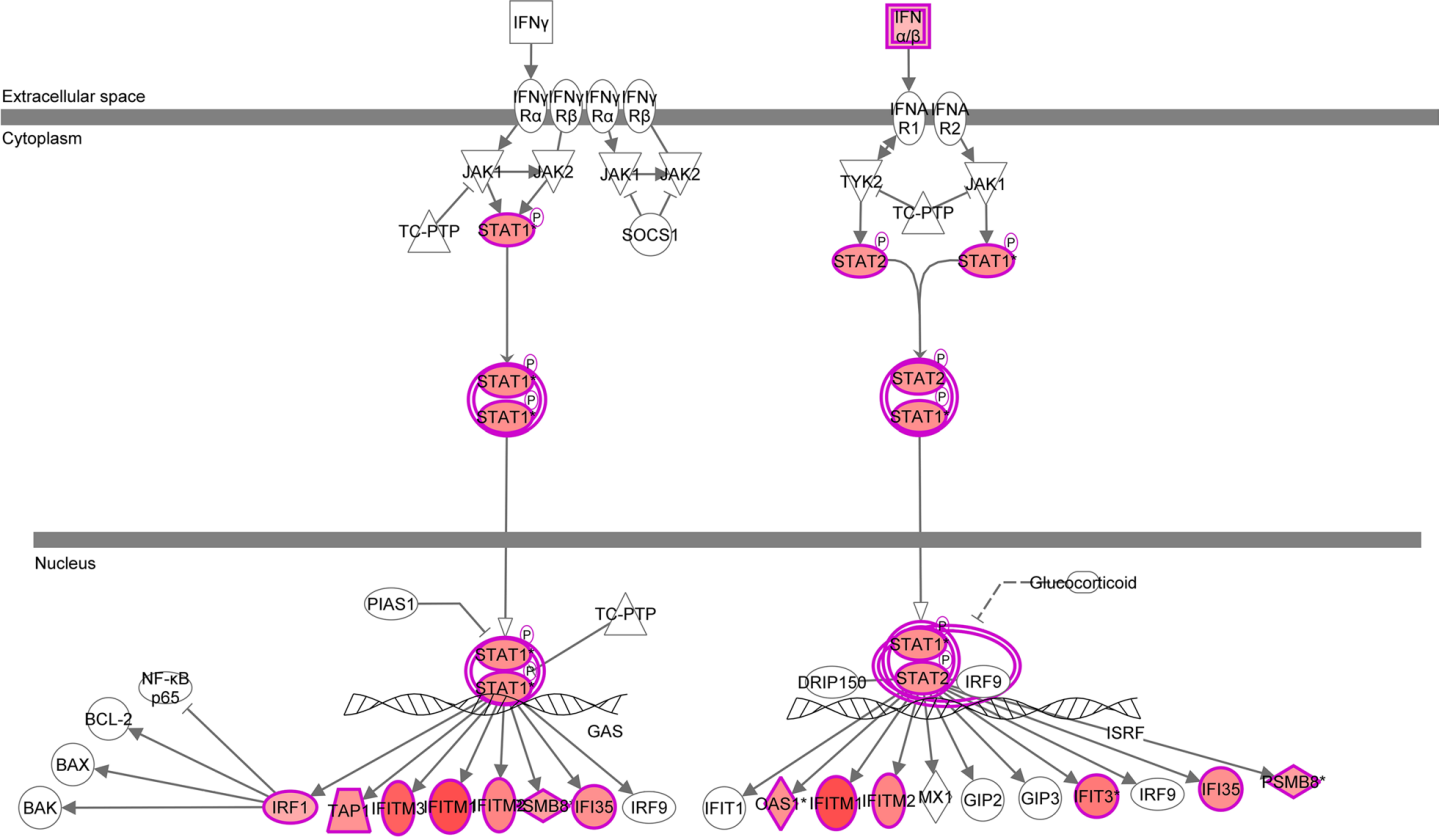
The functional network of the interferon signaling pathway by IPA. Genes that were significantly up-regulated in BNC2-transfected A549 cells are shown in *red*. The intensity of *red* corresponds to an increase in fold change

Subsequently, we analyzed which gene networks and functional pathways are influenced by BNC2 in A549 cells using 2 different analysis programs, IPA and g:Profiler. IPA pathway analysis software identified 148 statistically significant canonical pathways, of which the top 15 enriched signaling pathways with a *p* value <10^−5^ are shown in Additional file 2. The top three signaling pathways by IPA were interferon signaling, antigen presentation and the activation of IRF by cytosolic pattern recognition receptors. The signaling pathway that was affected the most, interferon signaling, and associated genes are shown in Fig. 3. Among gene regulatory networks, three networks, each consisting at least 40% of the affected genes, were identified by IPA. These three networks were associated with the inflammatory response, infectious diseases, immunological diseases and dermatological diseases and conditions (Additional file 3).

G:Profiler analysis revealed 188 statistically significant functional groups, of which over 100 had a *p* value less than 10^−5^. The top 20 BNC2-influenced functional groups are listed in Additional file 4. The most significant overlap was determined for the type I interferon signaling pathway, the cellular response to type I interferon and the cytokine-mediated signaling pathway.

### BNC2 induces the expression of ISGs with anti-cancerous properties

**Fig. 4:**
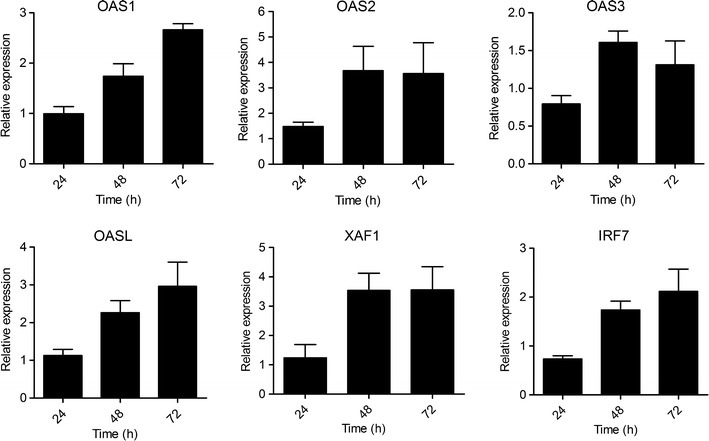
The over-expression of BNC2 induces the expression of ISGs associated with the repression of cancer development. Human A549 cells were transfected with either BNC2 or the control. Data represent the mean ± SEM of four transfections

Several ISGs, such as *OAS* family members and the tumor suppressors *XAF1* and *IRF7,* have been shown to play crucial roles in counteracting cancer progression, and their increased expression is associated with the inhibition of cell growth and the promotion of the apoptosis of cancer cell lines [40–43]. Concordantly, the reduced expression of *XAF1* and *OAS* family members has been observed in several cancer cell lines [44–47]. Our microarray data analysis revealed (Additional file 1) and RT-qPCR confirmed (Fig. 4) the increased mRNA expression of all of the *OAS* gene family members (OAS1, OAS2, OAS3, OASL) and XAF1 and IRF7 in the lung cancer cell line A549 after transfection with BNC2. Notably, the increased expression of the studied ISGs was persistent and could be detected 72 h after the transfection of the A549 cells with BNC2.

## Discussion

Lung cancer is a leading cause of cancer-related death worldwide [48]. Although improvements in molecular diagnostics and targeted therapies have been achieved in recent decades, the average 5-year survival rate for lung cancer is still below 20% [3]. New therapeutic targets are eagerly needed for this disease. In the current study, we demonstrate that human BNC2 is down-regulated in the adenocarcinomic alveolar epithelial cell line A549 and in SCC tissue compared to non-cancerous cells and tissue, respectively. The transfection of BNC2 to A549 cells led to the up-regulation of numerous ISGs, of which a subset (*XAF1, IRF7, OAS* family) is known to inhibit cancer growth and promote the apoptosis of cancer cells.

BNC2 was discovered as a gene with a similar domain structure as basonuclin 1, with a serine-rich region, nuclear localization signal (NLS) and three pairs of distinct C_2_H_2_ zinc fingers [20]. BNC2 is evolutionarily conserved in vertebrates: there is a remarkable conservation of the amino acid sequence of BNC2 across species as distant as the zebrafish, chicken, and mammals. The level of similarity of amino acids between human and mouse BNC2 is 97% [20, 21].

Early studies suggested that BNC2 might act as a transcription regulator [19, 20]. Later, it was proposed that BNC2 has a function in RNA processing [21] and may regulate the expression of genes essential for the development of craniofacial bones [49]. Multiple studies have demonstrated the down-regulation of BNC2 in numerous cancers [31–33]. Akagi and colleagues detected the decreased expression of BNC2 mRNA in esophageal adenocarcinoma cells and showed that the stable expression of BNC2 caused the growth arrest of tumor cells, which suggests that BNC2 is a tumor suppressor [31, 32]. Our results show that BNC2 was significantly down-regulated in the lung adenocarcinoma cell line A549 compared to the human normal bronchial epithelial cell line BEAS-2B, as well as in lung tumor tissue compared to non-tumor tissue. In addition, we also show that the over-expression of BNC2 inhibits the proliferation of A549 cells. Thus, our data are in line with previous studies that report the down-regulation of the *BNC2* gene in cancers of epithelial origin and indicate that BNC2 has a tumor-suppressive function.

Microarray technologies have been intensively used in cancer research [50–53] and are useful to profile gene expression patterns to facilitate diagnosis, predict the response to therapy, find new biomarkers and examine the development of drug resistance in cancer [54–56].

Microarray data from A549 cells transfected with BNC2 show the relationship of BNC2 with the modulation of immune system. Increased BNC2 expression in Th22 cells compared to other T cell subsets [57] and the suppression of NF-κB basal activity in HEK293 cells [58] have been reported previously. We determined the relationship of BNC2 with immune regulation with two different pathway analysis programs: IPA and G:profiler, which both revealed that the increased expression of BNC2 primarily affects genes associated with the interferon signaling pathway. Several ISGs with increased expression in BNC2-transfected cells have been associated with the restriction of tumor growth and development. For example, *XAF1* has been shown to inhibit proliferation and to induce the apoptosis of cancer cells as it negatively regulates the caspase-inhibiting activity of XIAP [42, 47]. Along with *XAF1*, we discovered the up-regulation of a subset of genes with the capacity to inhibit cell proliferation and to stimulate cancer cells to undergo apoptosis (*IRFs, IFIT1*-*3, ISG12a, IFITM* and the OAS family members) [59–61].

The use of interferons (IFNs) could be a potential strategy in the treatment of lung cancer [8]. Type I IFNs (the IFN-α family and IFN-β) have been used with some success for the treatment of different cancers, including hematological malignancies and solid tumors [62–65]. Type II IFN, IFN-γ, also has antitumor effects in various types of cancers [66, 67]. In addition to in vitro studies, several pre-clinical and clinical in vivo studies demonstrate the efficacy of type I IFNs alone or in combination with other treatments in cancer therapy [68–74].

Thus, our results suggest that BNC2 has the capacity to increase the expression of IFN-regulated genes and thereby act as a tumor suppressor gene in lung epithelial cells.

## Conclusion

Our results suggest that BNC2 is a tumor suppressor gene with reduced expression in lung cancer cells and with the capacity to inhibit cell proliferation and to up-regulate IFN-regulated genes.

## Additional files

**Additional file 1.** Differentially expressed genes by microarray analysis.

**Additional file 2.** Top 15 BNC2-influenced IPA pathways.

**Additional file 3.** BNC2-influenced genes in immune system-related gene networks. Genes that were significantly up-regulated are shown in red and genes that were down-regulated in green. The intensity of the color corresponds to an increase in fold change. A. BNC2-influenced genes in the inflammatory response gene network. B. BNC2-influenced genes in dermatological diseases and conditions, infectious disease and endocrine system disorder-associated gene networks. C. BNC2-influenced genes in antimicrobial response-associated gene networks.

**Additional file 4.** Top 20 canonical pathways identified by g:Profiler analysis.

## Abbreviations

BNC2: basonuclin 2
A549: adenocarcinomic human alveolar basal epithelial cell line
BEAS-2B: human normal lung epithelial cell line
NSCLC: non-small cell lung cancer
IFN: interferon
XAF1: XIAP associated factor-1
OAS: 2,5-oligoadenylate synthetase
CTLA-4: cytotoxic T-lymphocyte antigen-4
PD-1: programmed cell death-1 protein receptor
IRF: interferon regulatory factor
ISG: interferon stimulated gene
MX: myxovirus resistance protein
DAPK: death activating protein kinase
HaCaT: aneuploid immortal keratinocyte cell line from adult human skin
HeLa: cervical cancer cell line
HEK293: human embryonic kidney cell line
SCC: squamous cell carcinoma
ESD: esterase D
FDR: false discovery rate
IFITM: interferon-induced transmembrane protein
NLS: nuclear localization signal
NF-κB: nuclear factor kappa-light-chain-enhancer of activated B cells
IFIT: interferon Induced Protein with tetratricopeptide repeats
XIAP: X-linked inhibitor of apoptosis protein activator
ATP: adenosine triphosphate
PKR: protein kinase R
