# Supplementary material for: Human basonuclin 2 up-regulates a cascade set of interferon-stimulated genes with anti-cancerous properties in a lung cancer model
Source: Cancer Cell Int. 2017 Feb 6;17:18. doi: 10.1186/s12935-017-0394-x (PMC5294813; doi:10.1186/s12935-017-0394-x)
Supplement: Supplementary file 1 — Additional file 1. Differentially expressed genes by microarray analysis. [file 12935_2017_394_MOESM1_ESM.docx]

**Additional File 1: Table S1. Differentially expressed genes by microarray analysis.^a^**

| **Probe_ID** | **gene Symbol** | **gene Name** | **Fold Change** |
| --- | --- | --- | --- |
| ILMN_1674063 | OAS2 | 2'-5'-oligoadenylate synthetase 2, 69/71kDa | 3.136 |
| ILMN_1801246 | IFITM1 | interferon induced transmembrane protein 1 | 2.719 |
| ILMN_2058782 | IFI27 | interferon, alpha-inducible protein 27 | 2.661 |
| ILMN_1805750 | IFITM3 | interferon induced transmembrane protein 3 | 2.426 |
| ILMN_1674811 | OASL | 2'-5'-oligoadenylate synthetase-like | 2.133 |
| ILMN_1659913 | ISG20 | interferon stimulated exonuclease gene 20kDa | 2.102 |
| ILMN_1739428 | IFIT2 | interferon-induced protein with tetratricopeptide repeats 2 | 2.065 |
| ILMN_2239754 | IFIT3 | interferon-induced protein with tetratricopeptide repeats 3 | 2.049 |
| ILMN_2347798 | IFI6 | interferon, alpha-inducible protein 6 | 2.021 |
| ILMN_1701789 | IFIT3 | interferon-induced protein with tetratricopeptide repeats 3 | 1.914 |
| ILMN_1736729 | OAS2 | 2'-5'-oligoadenylate synthetase 2, 69/71kDa | 1.901 |
| ILMN_1729749 | HERC5 | HECT and RLD domain containing E3 ubiquitin protein ligase 5 | 1.890 |
| ILMN_1673352 | IFITM2 | interferon induced transmembrane protein 2 | 1.887 |
| ILMN_1781373 | IFIH1 | interferon induced with helicase C domain 1 | 1.809 |
| ILMN_1723912 | IFI44L | interferon-induced protein 44-like | 1.794 |
| ILMN_1778401 | HLA-B | major histocompatibility complex, class I, B | 1.790 |
| ILMN_1769520 | UBE2L6 | ubiquitin-conjugating enzyme E2L 6 | 1.745 |
| ILMN_1742618 | XAF1 | XIAP associated factor 1 | 1.731 |
| ILMN_2352121 | NT5C3A | 5'-nucleotidase, cytosolic IIIA | 1.725 |
| ILMN_2415144 | SP110 | SP110 nuclear body protein | 1.721 |
| ILMN_1690105 | STAT1 | signal transducer and activator of transcription 1, 91kDa | 1.710 |
| ILMN_1745374 | IFI35 | interferon-induced protein 35 | 1.702 |
| ILMN_1769734 | NT5C3A | 5'-nucleotidase, cytosolic IIIA | 1.692 |
| ILMN_1760062 | IFI44 | interferon-induced protein 44 | 1.688 |
| ILMN_2410826 | OAS1 | 2'-5'-oligoadenylate synthetase 1, 40/46kDa | 1.687 |
| ILMN_2098126 | CCL5 | chemokine (C-C motif) ligand 5 | 1.677 |
| ILMN_1751079 | TAP1 | transporter 1, ATP-binding cassette, sub-family B (MDR/TAP) | 1.658 |
| ILMN_1675640 | OAS1 | 2'-5'-oligoadenylate synthetase 1, 40/46kDa | 1.651 |
| ILMN_1690921 | STAT2 | signal transducer and activator of transcription 2, 113kDa | 1.619 |
| ILMN_1798181 | IRF7 | interferon regulatory factor 7 | 1.610 |
| ILMN_2231928 | MX2 | myxovirus (influenza virus) resistance 2 (mouse) | 1.597 |
| ILMN_1710937 | IFI16 | interferon, gamma-inducible protein 16 | 1.597 |
| ILMN_3240420 | USP18 | ubiquitin specific peptidase 18 | 1.592 |
| ILMN_1701114 | GBP1 | guanylate binding protein 1, interferon-inducible | 1.576 |
| ILMN_1710844 | PARP10 | poly (ADP-ribose) polymerase family, member 10 (PARP10) | 1.575 |
| ILMN_1659688 | LGALS3BP | lectin, galactoside-binding, soluble, 3 binding protein | 1.572 |
| ILMN_1731418 | SP110 | SP110 nuclear body protein | 1.571 |
| ILMN_1658247 | OAS1 | 2'-5'-oligoadenylate synthetase 1, 40/46kDa | 1.570 |
| ILMN_1731224 | PARP9 | poly (ADP-ribose) polymerase family, member 9 | 1.543 |
| ILMN_1718558 | PARP12 | poly (ADP-ribose) polymerase family, member 12 | 1.541 |
| ILMN_2349061 | IRF7 | interferon regulatory factor 7 | 1.539 |
| ILMN_3259146 | BST2 | bone marrow stromal cell antigen 2 | 1.538 |
| ILMN_1765258 | HLA-E | major histocompatibility complex, class I, E | 1.536 |
| ILMN_1723480 | BST2 | bone marrow stromal cell antigen 2 | 1.522 |
| ILMN_1773352 | CCL5 | chemokine (C-C motif) ligand 5 | 1.521 |
| ILMN_1779252 | TRIM22 | tripartite motif containing 22 | 1.520 |
| ILMN_1704972 | TRIM5 | tripartite motif containing 5 | 1.519 |
| ILMN_2130441 | HLA-H | major histocompatibility complex, class I, H (pseudogene) | 1.519 |
| ILMN_2262044 | PARP10 | poly (ADP-ribose) polymerase family, member 10 | 1.511 |
| ILMN_1703108 | UBE2L6 | ubiquitin-conjugating enzyme E2L 6 | 1.509 |
| ILMN_1672661 | SP110 | SP110 nuclear body protein | 1.508 |
| ILMN_1777325 | STAT1 | signal transducer and activator of transcription 1, 91kDa | 1.504 |
| ILMN_1755173 | PLEKHA4 | pleckstrin homology domain containing, family A (phosphoinositide binding specific) member 4 | 1.503 |
| ILMN_2148785 | GBP1 | guanylate binding protein 1, interferon-inducible | 1.501 |
| ILMN_1677198 | C1R | complement component 1, r subcomponent | 1.497 |
| ILMN_2370573 | XAF1 | XIAP associated factor 1 | 1.497 |
| ILMN_1721411 | PARP10 | poly (ADP-ribose) polymerase family, member 10 (PARP10) | 1.496 |
| ILMN_1695404 | LY6E | lymphocyte antigen 6 complex, locus E | 1.481 |
| ILMN_1781626 | C1S | complement component 1, s subcomponent | 1.480 |
| ILMN_1745397 | OAS3 | 2'-5'-oligoadenylate synthetase 3, 100kDa | 1.477 |
| ILMN_1691364 | STAT1 | signal transducer and activator of transcription 1, 91kDa | 1.463 |
| ILMN_2390299 | PSMB8 | proteasome (prosome, macropain) subunit, beta type, 8 | 1.462 |
| ILMN_1705241 | TDRD7 | tudor domain containing 7 | 1.434 |
| ILMN_1814305 | SAMD9 | sterile alpha motif domain containing 9 | 1.433 |
| ILMN_2248970 | OAS2 | 2'-5'-oligoadenylate synthetase 2, 69/71kDa | 1.433 |
| ILMN_1797001 | DDX58 | DEAD (Asp-Glu-Ala-Asp) box polypeptide 58 | 1.432 |
| ILMN_1683792 | LAP3 | leucine aminopeptidase 3 | 1.427 |
| ILMN_1750321 | TRANK1 | tetratricopeptide repeat and ankyrin repeat containing 1 | 1.421 |
| ILMN_1764109 | C1R | complement component 1, r subcomponent | 1.420 |
| ILMN_1705750 | TGM2 | transglutaminase 2 | 1.418 |
| ILMN_1664543 | IFIT3 | interferon-induced protein with tetratricopeptide repeats 3 | 1.403 |
| ILMN_1691731 | PARP14 | poly (ADP-ribose) polymerase family, member 14 | 1.402 |
| ILMN_1774287 | CFB | complement factor B | 1.401 |
| ILMN_2053527 | PARP9 | poly (ADP-ribose) polymerase family, member 9 | 1.398 |
| ILMN_2289093 | RNF213 | ring finger protein 213 | 1.389 |
| ILMN_1799467 | SAMD9L | sterile alpha motif domain containing 9-like | 1.388 |
| ILMN_1783621 | CMPK2 | cytidine monophosphate (UMP-CMP) kinase 2, mitochondrial | 1.384 |
| ILMN_1795181 | DDX60 | DEAD (Asp-Glu-Ala-Asp) box polypeptide 60 | 1.379 |
| ILMN_1690241 | BATF2 | basic leucine zipper transcription factor, ATF-like 2 | 1.378 |
| ILMN_2376108 | PSMB9 | proteasome (prosome, macropain) subunit, beta type, 9 | 1.376 |
| ILMN_1701613 | RARRES3 | retinoic acid receptor responder (tazarotene induced) 3 | 1.376 |
| ILMN_1678054 | TRIM21 | tripartite motif containing 21 | 1.375 |
| ILMN_1738523 | MYD88 | myeloid differentiation primary response 88 | 1.362 |
| ILMN_2388547 | EPSTI1 | epithelial stromal interaction 1 (breast) | 1.360 |
| ILMN_2186806 | HLA-F | major histocompatibility complex, class I, F | 1.344 |
| ILMN_2170814 | LAMP3 | lysosomal-associated membrane protein 3 | 1.344 |
| ILMN_3243928 | DDX60L | DEAD (Asp-Glu-Ala-Asp) box polypeptide 60-like | 1.341 |
| ILMN_1767006 | PSMB8 | proteasome (prosome, macropain) subunit, beta type, 8 | 1.335 |
| ILMN_1747195 | PSMB8 | proteasome (prosome, macropain) subunit, beta type, 8 | 1.330 |
| ILMN_2390162 | PHF11 | PHD finger protein 11 | 1.326 |
| ILMN_2203950 | HLA-A | major histocompatibility complex, class I, A | 1.325 |
| ILMN_2390586 | SP100 | SP100 nuclear antigen | 1.316 |
| ILMN_1681721 | OASL | 2'-5'-oligoadenylate synthetase-like | 1.312 |
| ILMN_2349393 | MDK | midkine (neurite growth-promoting factor 2) | 1.308 |
| ILMN_1762861 | HLA-F | major histocompatibility complex, class I, F | 1.305 |
| ILMN_1653466 | HES4 | hairy and enhancer of split 4 (Drosophila) | 1.304 |
| ILMN_1810608 | PNPT1 | polyribonucleotide nucleotidyltransferase 1 | 1.300 |
| ILMN_1708375 | IRF1 | interferon regulatory factor 1 | 1.299 |
| ILMN_1740466 | FAM46A | family with sequence similarity 46, member A | 1.296 |
| ILMN_1750400 | C19orf66 | chromosome 19 open reading frame 66 | 1.286 |
| ILMN_1751330 | RBCK1 | RanBP-type and C3HC4-type zinc finger containing 1 | 1.284 |
| ILMN_1777565 | TAP2 | transporter 2, ATP-binding cassette, sub-family B (MDR/TAP) | 1.283 |
| ILMN_2165753 | HLA-A29.1 | Homo sapiens major histocompatibility complex class I HLA-A29.1 (HLA-A29.1) | 1.282 |
| ILMN_1745242 | PLSCR1 | phospholipid scramblase 1 | 1.281 |
| ILMN_1654639 | HERC6 | HECT and RLD domain containing E3 ubiquitin protein ligase family member 6 | 1.279 |
| ILMN_2181445 | BCL2L13 | BCL2-like 13 (apoptosis facilitator) | 1.271 |
| ILMN_1776723 | PHF11 | PHD finger protein 11 | 1.269 |
| ILMN_2041190 | F2RL1 | coagulation factor II (thrombin) receptor-like 1 | 1.249 |
| ILMN_1721113 | HLA-C | major histocompatibility complex, class I, C | 1.242 |
| ILMN_1745807 | TMEM62 | transmembrane protein 62 | 1.239 |
| ILMN_2267914 | CD68 | CD68 molecule | 1.239 |
| ILMN_1683678 | SPATS2L | spermatogenesis associated, serine-rich 2-like | 1.236 |
| ILMN_3295494 | LAP3P2 | leucine aminopeptidase 3 pseudogene 2 | 1.234 |
| ILMN_1801307 | TNFSF10 | tumor necrosis factor (ligand) superfamily, member 10 | 1.233 |
| ILMN_1790472 | SLC25A28 | solute carrier family 25 (mitochondrial iron transporter), member 28 | 1.226 |
| ILMN_1803945 | HCP5 | Homo sapiens HLA complex P5 (HCP5), mRNA. | 1.222 |
| ILMN_1804396 | IRF2BPL | interferon regulatory factor 2 binding protein-like | 1.208 |
| ILMN_1736863 | TMEM140 | transmembrane protein 140 | 1.207 |
| ILMN_1690365 | USP41 | ubiquitin specific peptidase 41 | 1.206 |
| ILMN_2404665 | TRIM5 | tripartite motif containing 5 | 1.203 |
| ILMN_2284998 | SP100 | SP100 nuclear antigen | 1.202 |
| ILMN_1666078 | HLA-H | major histocompatibility complex, class I, H (pseudogene) | 1.196 |
| ILMN_1689456 | ZBTB20 | zinc finger and BTB domain containing 20 | 1.194 |
| ILMN_1657871 | RSAD2 | radical S-adenosyl methionine domain containing 2 | 1.189 |
| ILMN_1682336 | MASTL | microtubule associated serine/threonine kinase-like | 1.186 |
| ILMN_1749722 | RNF213 | ring finger protein 213 | 1.184 |
| ILMN_1690920 | SP100 | SP100 nuclear antigen | 1.179 |
| ILMN_1845037 | TRIM69 | tripartite motif containing 69 | 1.175 |
| ILMN_1724145 | CBX4 | chromobox homolog 4 | 1.175 |
| ILMN_1753805 | PRKD2 | protein kinase D2 | 1.174 |
| ILMN_1664010 | ELF1 | E74-like factor 1 (ets domain transcription factor) | 1.173 |
| ILMN_1690939 | TYMP | thymidine phosphorylase | 1.170 |
| ILMN_1813455 | SP110 | SP110 nuclear body protein | 1.168 |
| ILMN_3297126 | TYMP | thymidine phosphorylase | 1.166 |
| ILMN_2066858 | TNFSF13B | tumor necrosis factor (ligand) superfamily, member 13b | 1.164 |
| ILMN_1709333 | OAS2 | 2'-5'-oligoadenylate synthetase 2, 69/71kDa | 1.163 |
| ILMN_2149624 | IFNL1 | interferon, lambda 1 | 1.163 |
| ILMN_1725700 | MOV10 | Mov10, Moloney leukemia virus 10, homolog (mouse) | 1.162 |
| ILMN_2184262 | OAS3 | 2'-5'-oligoadenylate synthetase 3, 100kDa | 1.160 |
| ILMN_1720083 | EHD4 | EH-domain containing 4 | 1.159 |
| ILMN_1712046 | CPXM1 | carboxypeptidase X (M14 family), member 1 | 1.158 |
| ILMN_1782487 | GBP1P1 | guanylate binding protein 1, interferon-inducible pseudogene 1 | 1.156 |
| ILMN_1771385 | GBP4 | guanylate binding protein 4 | 1.154 |
| ILMN_1727045 | RASGRP3 | RAS guanyl releasing protein 3 (calcium and DAG-regulated) | 1.146 |
| ILMN_1814686 | BCMO1 | beta-carotene 15,15'-monooxygenase 1 | 1.142 |
| ILMN_2085862 | SLC15A3 | solute carrier family 15 (oligopeptide transporter), member 3 | 1.142 |
| ILMN_2205999 | OSMR | oncostatin M receptor | 1.138 |
| ILMN_3239445 | ZBTB42 | zinc finger and BTB domain containing 42 | 1.137 |
| ILMN_1794612 | UBA7 | ubiquitin-like modifier activating enzyme 7 | 1.137 |
| ILMN_1665291 | NUB1 | negative regulator of ubiquitin-like proteins 1 | 1.134 |
| ILMN_1815134 | PI4K2B | phosphatidylinositol 4-kinase type 2 beta | 1.133 |
| ILMN_1697971 | TRIM38 | tripartite motif containing 38 | 1.132 |
| ILMN_1738712 | GPR180 | G protein-coupled receptor 180 | 1.132 |
| ILMN_1668345 | OAF | OAF homolog (Drosophila) | 1.132 |
| ILMN_1682081 | RNF19B | ring finger protein 19B | 1.130 |
| ILMN_1716704 | NLRC5 | NLR family, CARD domain containing 5 | 1.129 |
| ILMN_1672606 | OAS1 | 2'-5'-oligoadenylate synthetase 1, 40/46kDa | 1.128 |
| ILMN_3246953 | CMTR1 | cap methyltransferase 1 | 1.126 |
| ILMN_1835092 | IFI44L | interferon-induced protein 44-like | 1.123 |
| ILMN_1784110 | CDK18 | cyclin-dependent kinase 18 | 1.121 |
| ILMN_1701204 | VEGFC | vascular endothelial growth factor C | 1.117 |
| ILMN_1759250 | TAP2 | transporter 2, ATP-binding cassette, sub-family B (MDR/TAP) | 1.112 |
| ILMN_1662302 | IFNL2 | interferon, lambda 2 | 1.111 |
| ILMN_1659960 | IL4I1 | interleukin 4 induced 1 | 1.110 |
| ILMN_2326509 | CASP1 | caspase 1, apoptosis-related cysteine peptidase | 1.106 |
| ILMN_1756992 | MUC1 | mucin 1, cell surface associated | 1.105 |
| ILMN_1758418 | TNFSF13B | tumor necrosis factor (ligand) superfamily, member 13b | 1.095 |
| ILMN_1677603 | C1S | complement component 1, s subcomponent | 1.094 |
| ILMN_1676528 | BTN3A2 | butyrophilin, subfamily 3, member A2 | 1.094 |
| ILMN_1733680 | AZI2 | 5-azacytidine induced 2 | 1.093 |
| ILMN_1682245 | IFNB1 | interferon, beta 1, fibroblast | 1.091 |
| ILMN_2325337 | APOL2 | apolipoprotein L, 2 | 1.089 |
| ILMN_2170813 | LAMP3 | lysosomal-associated membrane protein 3 | 1.088 |
| ILMN_1678422 | DHX58 | DEXH (Asp-Glu-X-His) box polypeptide 58 | 1.087 |
| ILMN_2070044 | PPM1K | protein phosphatase, Mg2+/Mn2+ dependent, 1K | 1.085 |
| ILMN_1737599 | TRIM5 | tripartite motif containing 5 | 1.084 |
| ILMN_1758731 | CYP2J2 | cytochrome P450, family 2, subfamily J, polypeptide 2 | 1.084 |
| ILMN_2235975 | FXYD5 | FXYD domain containing ion transport regulator 5 | 1.077 |
| ILMN_1800078 | LMO2 | LIM domain only 2 (rhombotin-like 1) | 1.069 |
| ILMN_1797359 | TRIM78P | tripartite motif containing 78, pseudogene | 1.069 |
| ILMN_3230337 | PAXIP1-AS2 | PAXIP1 antisense RNA 2 | 1.057 |
| ILMN_1750800 | ACO1 | aconitase 1, soluble | -1.499 |
| ILMN_3248882 | DANCR | differentiation antagonizing non-protein coding RNA | -1.308 |
| ILMN_2188722 | GLS | glutaminase | -1.224 |
| ILMN_1708743 | NT5DC2 | 5'-nucleotidase domain containing 2 | -1.174 |
| ILMN_2182531 | TIMM21 | translocase of inner mitochondrial membrane 21 homolog (yeast) | -1.161 |
| ILMN_1748916 | TIMM21 | translocase of inner mitochondrial membrane 21 homolog (yeast) | -1.156 |
| ILMN_1756402 | TMEM177 | transmembrane protein 177 | -1.154 |
| ILMN_1783753 | TXNDC12 | thioredoxin domain containing 12 (endoplasmic reticulum) | -1.132 |
| ILMN_1690523 | LRRC20 | leucine rich repeat containing 20 | -1.119 |
| ILMN_1808757 | RPL37A | ribosomal protein L37a | -1.105 |
| ILMN_1867188 | LOC100293704 | serine/arginine repetitive matrix protein 3-like | -1.100 |
| ILMN_1814797 | SLC35F3 | solute carrier family 35, member F3 (SLC35F3) | -1.099 |
| ILMN_2368576 | UBA52 | ubiquitin A-52 residue ribosomal protein fusion product 1 | -1.093 |
| ILMN_1726666 | GPX3 | glutathione peroxidase 3 (plasma) | -1.071 |

^a^Significantly up- or downregulated transcripts with p < 0.05 are included in the table
