# Supplementary material for: Human basonuclin 2 up-regulates a cascade set of interferon-stimulated genes with anti-cancerous properties in a lung cancer model
Source: Cancer Cell Int. 2017 Feb 6;17:18. doi: 10.1186/s12935-017-0394-x (PMC5294813; doi:10.1186/s12935-017-0394-x)
Supplement: Supplementary file 2 — Additional file 2. Top 15 BNC2-influenced IPA pathways. [file 12935_2017_394_MOESM2_ESM.docx]

**Additional File 2: Table S2**. **Top15 BNC2-influenced** **IPA pathways**

| **Ingenuity Canonical Pathways** | ***p*-value^a^** | **Ratio^b^** | **Overlapped genes** |
| --- | --- | --- | --- |
| Interferon Signaling | 3.23E-17 | 3,33E-01 (12/36) | IFIT3, IFITM3, OAS1, IFNB1, IFI35, STAT2, PSMB8, STAT1, TAP1, IFITM2, IRF1, IFITM1 |
| Antigen Presentation Pathway | 1.70E-13 | 2,7E-01 (10/37) | PSMB9, NLRC5, HLA-A, HLA-C, HLA-B, PSMB8, HLA-F, TAP1, TAP2, HLA-E |
| Activation of IRF by Cytosolic Pattern Recognition Receptors | 1.99E-08 | 1,33E-01 (8/60) | DHX58, IFIH1, IRF7, DDX58, IFNB1, STAT2, STAT1, IFIT2 |
| Role of Pattern Recognition Receptors in Recognition of Bacteria and Viruses | 3.03E-08 | 8,4E-02 (10/119) | IFIH1, OAS1, IRF7, OAS2, MYD88, DDX58, CASP1, IFNB1, CCL5, OAS3 |
| Communication between Innate and Adaptive Immune Cells | 2.40E-07 | 9,76E-02 (8/82) | HLA-A, HLA-C, HLA-B, IFNB1, CCL5, HLA-F, TNFSF13B, HLA-E |
| Retinoic acid Mediated Apoptosis Signaling | 3.60E-07 | 1,19E-01 (7/59) | PARP10, IFNB1, TNFSF10, PARP12, PARP9, IRF1, PARP14 |
| Type I Diabetes Mellitus Signaling | 1.74E-06 | 7,55E-02 (8/106) | HLA-A, HLA-C, MYD88, HLA-B, HLA-F, STAT1, IRF1, HLA-E |
| Crosstalk between Dendritic Cells and Natural Killer Cells | 5.99E-06 | 7,87E-02 (7/89) | HLA-A, HLA-C, HLA-B, IFNB1, TNFSF10, HLA-F, HLA-E |
| Neuroprotective Role of THOP1 in Alzheimer's Disease | 1.40E-05 | 1,25E-01 (5/40) | HLA-A, HLA-C, HLA-B, HLA-F, HLA-E |
| Role of RIG1-like Receptors in Antiviral Innate Immunity | 1.58E-05 | 1,22E-01 (5/41) | DHX58, IFIH1, IRF7, DDX58, IFNB1 |
| Autoimmune Thyroid Disease Signaling | 1.79E-05 | 1,19E-01 (5/42) | HLA-A, HLA-C, HLA-B, HLA-F, HLA-E |
| Graft-versus-Host Disease Signaling | 2.25E-05 | 1,14E-01 (5/44) | HLA-A, HLA-C, HLA-B, HLA-F, HLA-E |
| Protein Ubiquitination Pathway | 3.01E-05 | 3,94E-02 (10/254) | PSMB9, USP18, USP41, HLA-A, HLA-C, HLA-B, PSMB8, TAP1, UBE2L6, TAP2 |
| Allograft Rejection Signaling | 3.46E-05 | 1,04E-01 (5/48) | HLA-A, HLA-C, HLA-B, HLA-F, HLA-E |
| OX40 Signaling Pathway | 6.16E-05 | 9,26E-02 (5/54) | HLA-A, HLA-C, HLA-B, HLA-F, HLA-E |

^a^The *p*-value from Fisher exact test showing the significance of the overlap between BNC2-influenced genes and indicated canonical pathway

^b^the ratio of the overlap and the number of genes in the discovery set
