# Supplementary material for: Human basonuclin 2 up-regulates a cascade set of interferon-stimulated genes with anti-cancerous properties in a lung cancer model
Source: Cancer Cell Int. 2017 Feb 6;17:18. doi: 10.1186/s12935-017-0394-x (PMC5294813; doi:10.1186/s12935-017-0394-x)
Supplement: Supplementary file 3 — Additional file 3. BNC2-influenced genes in immune system-related gene networks. Genes that were significantly up-regulated are shown in red and genes that were down-regulated in green. The intensity of the color corresponds to an increase in fold change. A. BNC2-influenced genes in the inflammatory response gene network. B. BNC2-influenced genes in dermatological diseases and conditions, infectious disease and endocrine system disorder-associated gene networks. C. BNC2-influenced genes in antimicrobial response-associated gene networks. [file 12935_2017_394_MOESM3_ESM.pdf]

Supplementary Figure 1

A

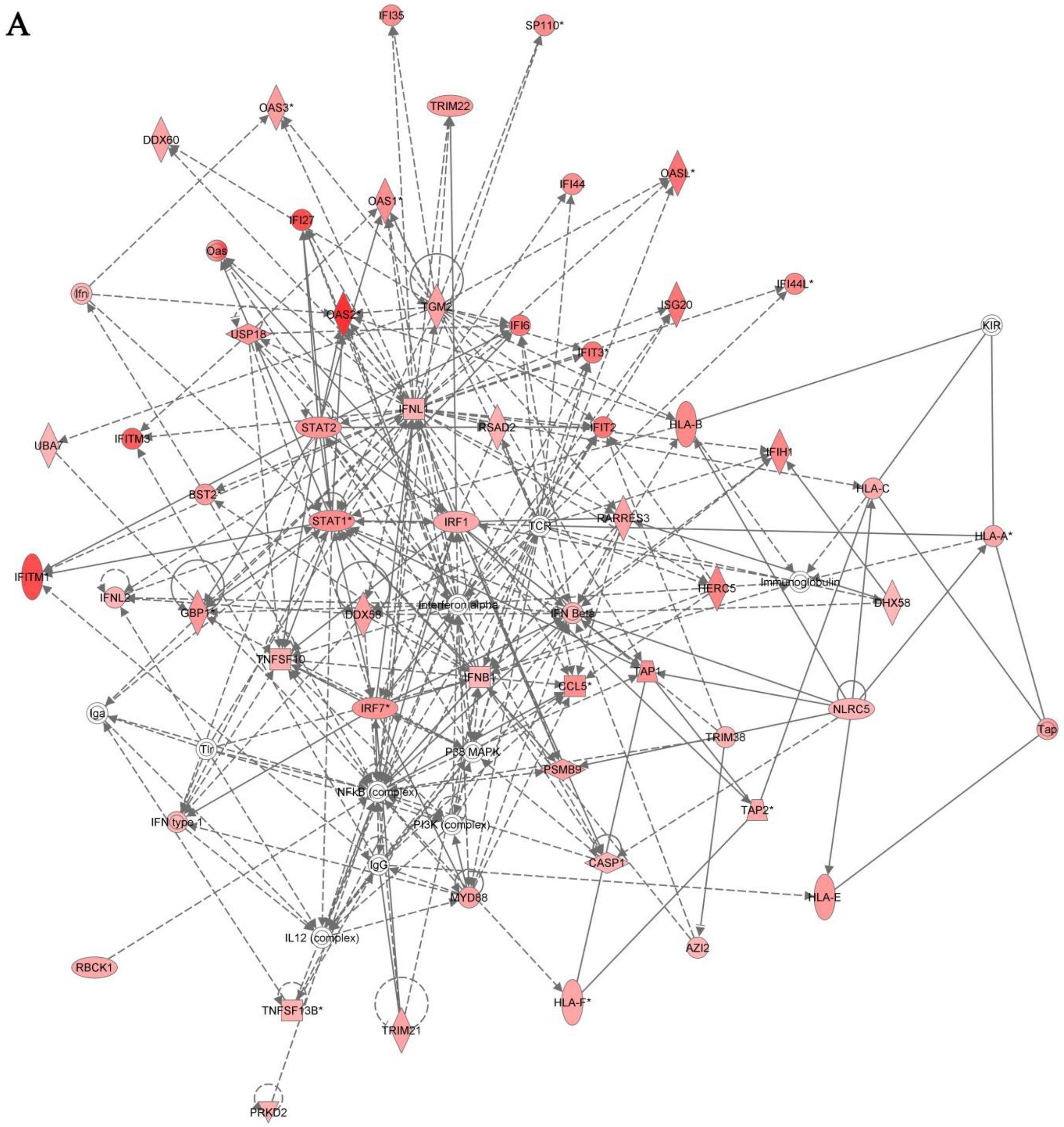

B

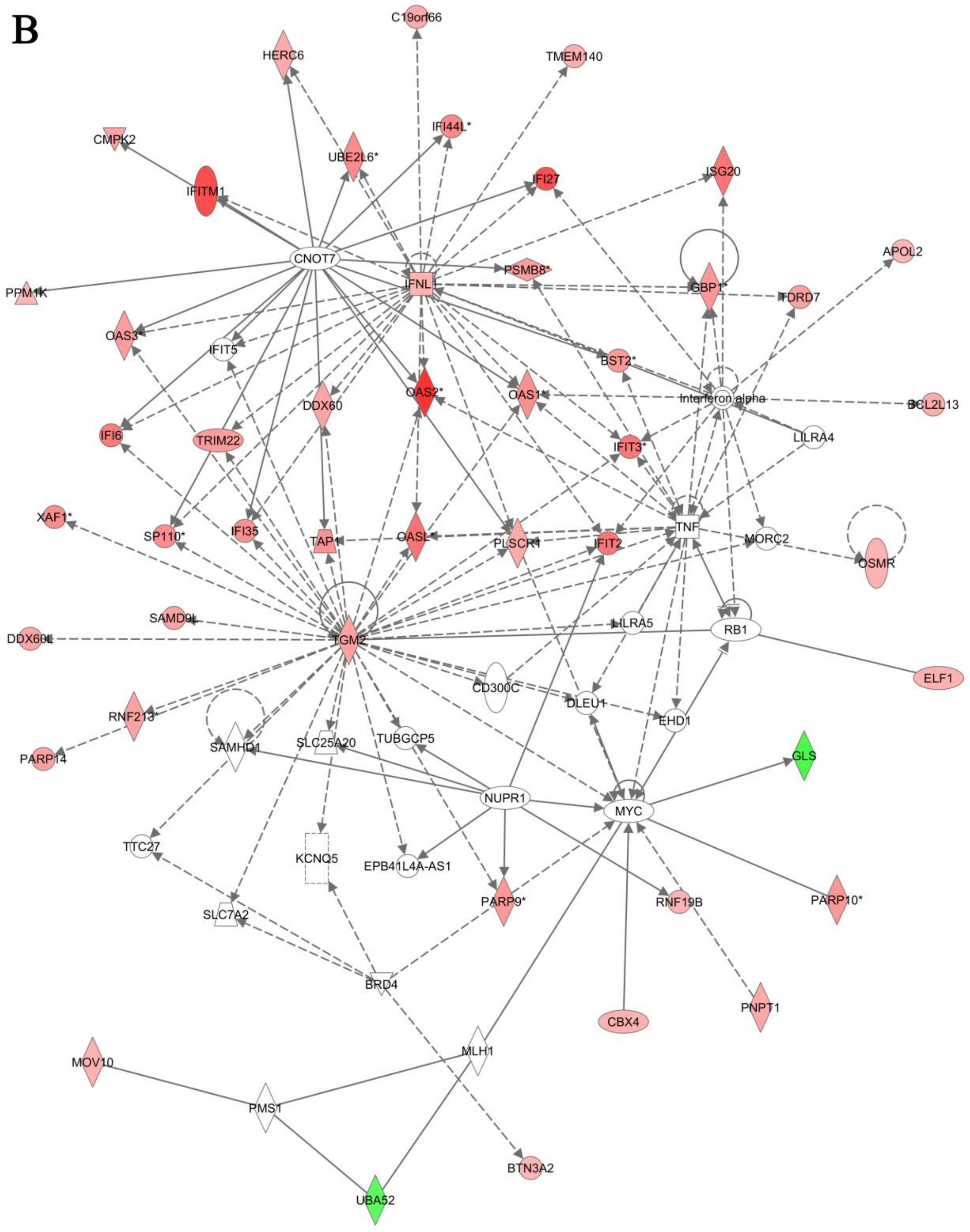
C19orf66
HERC6
TMEM140
IFI44L*
CMPK2
UBE2L6*
IFITM1
IFI27
ISG20
CNOT7
APOL2
PSMB8*
IFNL1
GBP1*
PPM1K
TDRD7
OAS3*
IFIT5
BST2*
OAS2*
OAS1*
DDX60
Interferon alpha
BCL2L13
IFI6
TRIM22
LILRA4
IFIT3*
XAF1*
SP110*
IFI35
TAP1
OASL*
PLSCR1
IFIT2
TNF
MORC2
OSMR
SAMD9L
LILRA5
DDX60L
TGM2
RB1
ELF1
CD300C
DLEU1
RNF213*
EHD1
SAMHD1
SLC25A20
TUBGCP5
GLS
PARP14
NUPR1
MYC
TTC27
KCNQ5
EPB41L4A-AS1
PARP9*
RNF19B
PARP10*
SLC7A2
BRD4
PNPT1
CBX4
MOV10
MLH1
PMS1
BTN3A2
UBA52

C

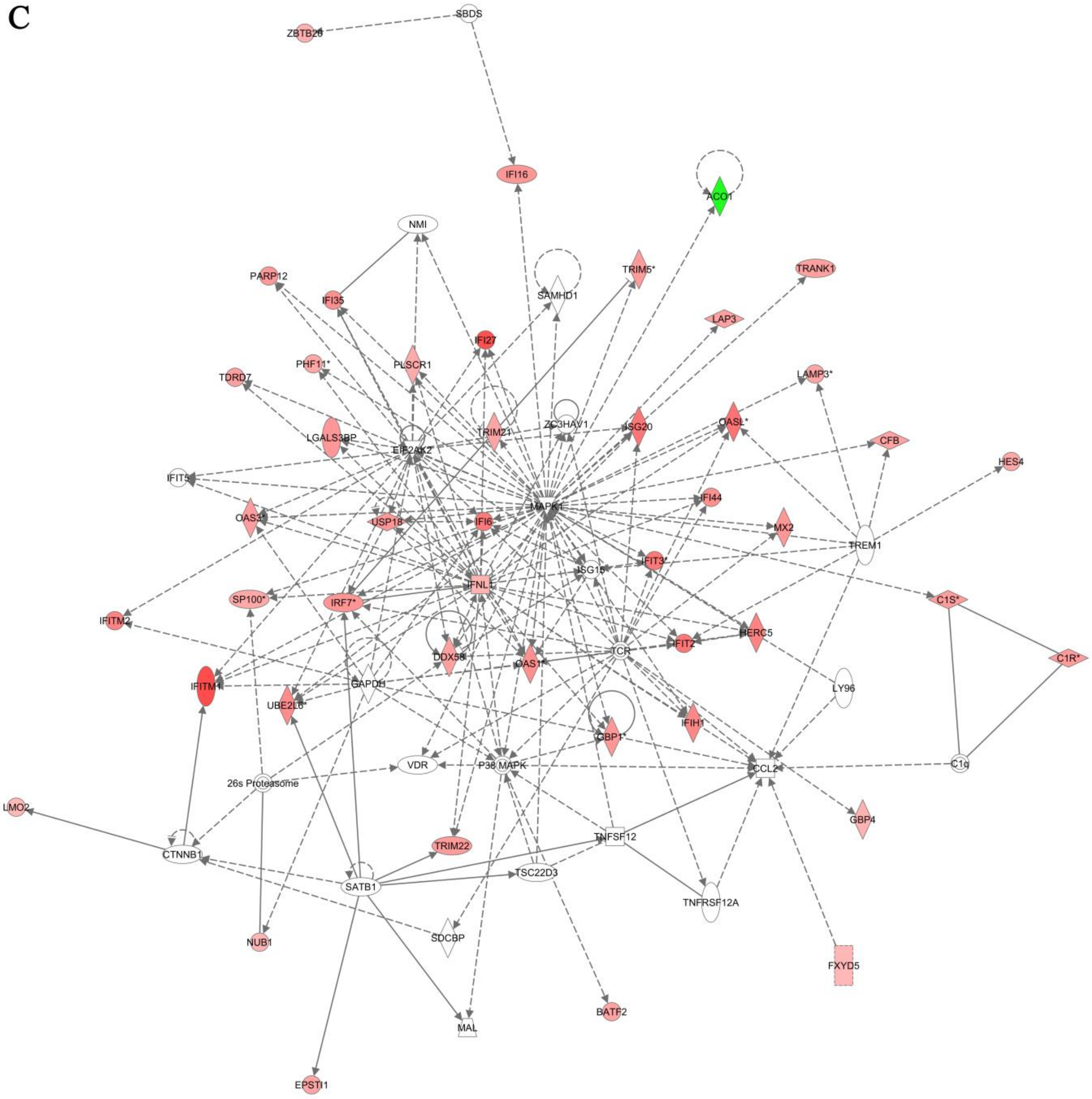

SBDS
ZBTB20
IFI16
ACO1
NMI
PARP12
IFI35
SAMHD1
TRIM5*
TRANK1
LAP3
IFI27
PHF11*
PLSCR1
TDRD7
LAMP3*
ZC3HAV1
LGALS3BP
TRIM21
ISG20
OASL*
EIF2AK2
CFB
IFIT5
HES4
MAPK1
IFI44
OAS3*
USP18
IFI6
MX2
TREM1
IFIT3*
ISG15*
IFNL1
C1S*
SP100*
IRF7*
IFITM2
HERC5
IFIT2
DDX58
TCR
C1R*
OAS1*
GAPDH
LY96
IFITM1
UBE2L6*
IFIH1
GBP1*
VDR
P38 MAPK
CCL2
C1q
26s Proteasome
LMO2
GBP4
TNFSF12
TRIM22
CTNNB1
TSC22D3
SATB1
TNFRSF12A
SDCBP
NUB1
FXYD5
BATF2
MAL
EPSTI1
