# Supplementary material for: Human basonuclin 2 up-regulates a cascade set of interferon-stimulated genes with anti-cancerous properties in a lung cancer model
Source: Cancer Cell Int. 2017 Feb 6;17:18. doi: 10.1186/s12935-017-0394-x (PMC5294813; doi:10.1186/s12935-017-0394-x)
Supplement: Supplementary file 4 — Additional file 4. Top 20 canonical pathways identified by g:Profiler analysis. [file 12935_2017_394_MOESM4_ESM.docx]

**Additional File 4: Table S3**. **Top 20 canonical pathways identified by g:Profiler analysis.**

| **Canonical Pathways and Gene Ontology groups** | ***p*-value** | **Molecules** |
| --- | --- | --- |
| Type I interferon signaling pathway | 1.02e-37 | OAS2, IFITM1, IFI27, IFITM3, OASL, ISG20, IFIT2, IFIT3, IFI6, IFITM2, XAF1, STAT1, IFI35, OAS1, STAT2, IRF7, MX2, USP18, HLA-E, OAS3, PSMB8, HLA-F, HLA-A, SP100, IRF1, HLA-C, NLRC5, IFNB1 |
| Cellular response to type I interferon | 1.58e-37 | OAS2, IFITM1, IFI27, IFITM3, OASL, ISG20, IFIT2, IFIT3, IFI6, IFITM2, XAF1, STAT1, IFI35, OAS1, STAT2, IRF7, MX2, USP18, HLA-E, OAS3, PSMB8, HLA-F, HLA-A, SP100, IRF1, HLA-C, NLRC5, IFNB1 |
| Response to type I interferon | 2.43e-37 | OAS2, IFITM1, IFI27, IFITM3, OASL, ISG20, IFIT2, IFIT3, IFI6, IFITM2, XAF1, STAT1, IFI35, OAS1, STAT2, IRF7, MX2, USP18, HLA-E, OAS3, PSMB8, HLA-F, HLA-A, SP100, IRF1, HLA-C, NLRC5, IFNB1 |
| Defense response to virus | 6.92e-35 | OAS2, IFITM1, IFITM3, OASL, ISG20, IFIT2, IFIT3, HERC5, IFITM2, IFIH1, IFI44L, STAT1, OAS1, STAT2, IRF7, MX2, IFI16, GBP1, BST2, TRIM22, TRIM5, OAS3, DDX58, DDX60, HLA-A, IRF1, PLSCR1, F2RL1, RSAD2, IFNL1, TRIM38, NLRC5, IFNL2, IFNB1, DHX58 |
| Response to virus | 6.09e-34 | OAS2, IFITM1, IFITM3, OASL, ISG20, IFIT2, IFIT3, HERC5, IFITM2, IFIH1, IFI44L, STAT1, IFI44, OAS1, CCL5, STAT2, IRF7, MX2, IFI16, GBP1, BST2, TRIM22, TRIM5, OAS3, DDX58, DDX60, MYD88, HLA-A, IRF1, PLSCR1, F2RL1, RSAD2, IFNL1, TRIM38, NLRC5, IFNL2, IFNB1, DHX58 |
| Defense response | 2.51e-33 | OAS2, IFITM1, IFI27, IFITM3, OASL, ISG20, IFIT2, IFIT3, IFI6, HERC5, IFITM2, IFIH1, IFI44L, UBE2L6, XAF1, STAT1, IFI35, OAS1, CCL5, TAP1, STAT2, IRF7, MX2, IFI16, USP18, GBP1, LGALS3BP, PARP9, HLA-E, BST2, TRIM22, TRIM5, C1R, C1S, OAS3, PSMB8, DDX58, TGM2, DDX60, BATF2, TRIM21, MYD88, HLA-F, HLA-A, SP100, MDK, IRF1, PLSCR1, F2RL1, HLA-C, HCP5, RSAD2, IFNL1, MOV10, OSMR, UBA7, NUB1, TRIM38, RNF19B, NLRC5, IFNL2, CASP1, IFNB1, APOL2, DHX58, UBA52 |
| Immune response | 3.66e-33 | OAS2, IFITM1, IFI27, IFITM3, OASL, ISG20, IFIT2, IFIT3, IFI6, HERC5, IFITM2, IFIH1, IFI44L, UBE2L6, XAF1, STAT1, IFI35, OAS1, CCL5, TAP1, STAT2, IRF7, MX2, IFI16, USP18, GBP1, PARP9, HLA-E, BST2, TRIM22, TRIM5, HLA-H, C1R, C1S, OAS3, PSMB8, DDX58, DDX60, TRIM21, MYD88, HLA-F, HLA-A, SP100, IRF1, RBCK1, PLSCR1, F2RL1, HLA-C, TNFSF10, RSAD2, PRKD2, ELF1, TNFSF13B, IFNL1, MOV10, UBA7, NUB1, RNF19B, NLRC5, CASP1, BTN3A2, IFNB1, DHX58, UBA52 |
| Innate immune response | 2.04e-32 | OAS2, IFITM1, IFI27, IFITM3, OASL, ISG20, IFIT2, IFIT3, IFI6, HERC5, IFITM2, IFIH1, UBE2L6, XAF1, STAT1, IFI35, OAS1, CCL5, STAT2, IRF7, MX2, IFI16, USP18, GBP1, PARP9, HLA-E, BST2, TRIM5, C1R, C1S, OAS3, PSMB8, DDX58, DDX60, TRIM21, MYD88, HLA-F, HLA-A, SP100, IRF1, PLSCR1, F2RL1, HLA-C, RSAD2, MOV10, UBA7, NUB1, RNF19B, NLRC5, CASP1, IFNB1, DHX58, UBA52 |
| Immune effector process | 2.76e-30 | OAS2, IFITM1, IFITM3, OASL, ISG20, IFIT2, IFIT3, HERC5, IFITM2, IFIH1, IFI44L, STAT1, OAS1, TAP1, STAT2, IRF7, MX2, IFI16, GBP1, HLA-E, BST2, TRIM22, TRIM5, HLA-H, C1R, C1S, OAS3, DDX58, DDX60, MYD88, HLA-F, HLA-A, IRF1, PLSCR1, F2RL1, HLA-C, RSAD2, IFNL1, TRIM38, RNF19B, NLRC5, IFNL2, BTN3A2, IFNB1, DHX58 |
| Cytokine-mediated signaling pathway | 2.23e-28 | OAS2, IFITM1, IFI27, IFITM3, OASL, ISG20, IFIT2, IFIT3, IFI6, HERC5, IFITM2, UBE2L6, XAF1, STAT1, IFI35, OAS1, CCL5, STAT2, IRF7, MX2, USP18, GBP1, HLA-E, OAS3, PSMB8, MYD88, HLA-F, HLA-A, SP100, IRF1, F2RL1, HLA-C, OSMR, UBA7, NLRC5, IFNB1, UBA52 |
| Defense response to other organism | 5.94e-28 | OAS2, IFITM1, IFITM3, OASL, ISG20, IFIT2, IFIT3, HERC5, IFITM2, IFIH1, IFI44L, STAT1, OAS1, STAT2, IRF7, MX2, IFI16, GBP1, BST2, TRIM22, TRIM5, OAS3, DDX58, DDX60, BATF2, MYD88, HLA-A, IRF1, PLSCR1, F2RL1, RSAD2, IFNL1, TRIM38, NLRC5, IFNL2, IFNB1, DHX58 |
| Immune system process | 1.92e-27 | OAS2, IFITM1, IFI27, IFITM3, OASL, ISG20, IFIT2, IFIT3, IFI6, HERC5, IFITM2, IFIH1, IFI44L, UBE2L6, XAF1, STAT1, IFI35, OAS1, CCL5, TAP1, STAT2, IRF7, MX2, IFI16, USP18, GBP1, PARP9, HLA-E, BST2, TRIM22, TRIM5, HLA-H, C1R, C1S, OAS3, PSMB8, DDX58, DDX60, BATF2, PSMB9, TRIM21, MYD88, HLA-F, LAMP3, HLA-A, SP100, IRF1, RBCK1, PLSCR1, F2RL1, HLA-C, TNFSF10, RSAD2, PRKD2, ELF1, TNFSF13B, IFNL1, MOV10, UBA7, NUB1, TRIM38, RNF19B, NLRC5, VEGFC, IFNL2, CASP1, BTN3A2, IFNB1, DHX58, LMO2, UBA52 |
| Response to cytokine | 8.18e-27 | OAS2, IFITM1, IFI27, IFITM3, OASL, ISG20, IFIT2, IFIT3, IFI6, HERC5, IFITM2, UBE2L6, XAF1, STAT1, IFI35, OAS1, CCL5, STAT2, IRF7, MX2, USP18, GBP1, PARP9, HLA-E, BST2, OAS3, PSMB8, MYD88, HLA-F, HLA-A, SP100, PNPT1, IRF1, PLSCR1, F2RL1, HLA-C, OSMR, UBA7, NUB1, NLRC5, IFNB1, UBA52 |
| Cellular response to cytokine stimulus | 3.93e-25 | OAS2, IFITM1, IFI27, IFITM3, OASL, ISG20, IFIT2, IFIT3, IFI6, HERC5, IFITM2, UBE2L6, XAF1, STAT1, IFI35, OAS1, CCL5, STAT2, IRF7, MX2, USP18, GBP1, HLA-E, OAS3, PSMB8, MYD88, HLA-F, HLA-A, SP100, PNPT1, IRF1, F2RL1, HLA-C, OSMR, UBA7, NLRC5, IFNB1, UBA52 |
| Response to external biotic stimulus | 2.59e-23 | OAS2, IFITM1, IFITM3, OASL, ISG20, IFIT2, IFIT3, HERC5, IFITM2, IFIH1, IFI44L, STAT1, IFI44, OAS1, CCL5, STAT2, IRF7, MX2, IFI16, GBP1, BST2, TRIM22, TRIM5, OAS3, DDX58, CMPK2, DDX60, BATF2, MYD88, HLA-A, IRF1, PLSCR1, F2RL1, RSAD2, IFNL1, TRIM38, NLRC5, IFNL2, CASP1, IFNB1, DHX58 |
| Response to other organism | 2.59e-23 | OAS2, IFITM1, IFITM3, OASL, ISG20, IFIT2, IFIT3, HERC5, IFITM2, IFIH1, IFI44L, STAT1, IFI44, OAS1, CCL5, STAT2, IRF7, MX2, IFI16, GBP1, BST2, TRIM22, TRIM5, OAS3, DDX58, CMPK2, DDX60, BATF2, MYD88, HLA-A, IRF1, PLSCR1, F2RL1, RSAD2, IFNL1, TRIM38, NLRC5, IFNL2, CASP1, IFNB1, DHX58 |
| Response to biotic stimulus | 1.32e-22 | OAS2, IFITM1, IFITM3, OASL, ISG20, IFIT2, IFIT3, HERC5, IFITM2, IFIH1, IFI44L, STAT1, IFI44, OAS1, CCL5, STAT2, IRF7, MX2, IFI16, GBP1, BST2, TRIM22, TRIM5, OAS3, DDX58, CMPK2, DDX60, BATF2, MYD88, HLA-A, IRF1, PLSCR1, F2RL1, RSAD2, IFNL1, TRIM38, NLRC5, IFNL2, CASP1, IFNB1, DHX58 |
| Negative regulation of multi-organism process | 1.24e-20 | IFITM1, IFITM3, OASL, ISG20, IFITM2, OAS1, CCL5, IFI16, PARP10, BST2, TRIM5, OAS3, TRIM21, MYD88, SP100, PLSCR1, RSAD2, TRIM38, IFNB1, DHX58 |
| Response to interferon-gamma | 2.51e-20 | OAS2, IFITM1, IFITM3, OASL, IFITM2, STAT1, OAS1, CCL5, IRF7, GBP1, PARP9, HLA-E, BST2, OAS3, HLA-F, HLA-A, SP100, IRF1, HLA-C, NUB1, NLRC5 |
| Negative regulation of viral process | 4.85e-19 | IFITM1, IFITM3, OASL, ISG20, IFITM2, OAS1, CCL5, IFI16, PARP10, BST2, TRIM5, OAS3, TRIM21, SP100, PLSCR1, RSAD2, IFNB1 |
